# A Right to Constructive Optimization: A Public Interest Approach to Recommender Systems in the Digital Services Act

**DOI:** 10.1007/s10603-025-09586-1

**Published:** 2025-03-28

**Authors:** L. Naudts, N. Helberger, M. Veale, M. Sax

**Affiliations:** 1 Institute for Information Law, University of Amsterdam, Amsterdam, Netherlands; 2 Centre for IT & IP Law, KU Leuven, Leuven, Belgium; 3 Faculty of Laws, University College London, London, UK

**Keywords:** Digital Services Act, Very large online platforms, Recommender systems, Iris Marion Young, Social justice, Fundamental rights, Artificial Intelligence, Automated Decisions, Algorithms, European Law

## Abstract

The technological promise of recommender systems should not be misused by those with decisional power over the infrastructural, data, and knowledge resources needed for their design. The ideal of personalization should not mask self-serving optimization. Instead, we propose that people, not only in their capacity as consumers but, more generally, as democratic citizens, have a legitimate claim to ensure that very large online platforms (or VLOPs) respect their interests within optimization processes through the content policy strategies and recommendation technologies they employ. To this end, this paper argues for, and develops, a right to constructive optimization that promotes people’s effective enjoyment of fundamental rights and civic values in digital settings. The argument is structured as follows. First, the paper strengthens the claim that the largest online platforms perform a public function (although this is not the only way such functions can be performed). Second, drawing from the philosophy of Iris Marion Young, the paper identifies self-determination and self-development as key values recommenders should promote as part of this crucial function under conditions of inclusivity, political equality, reasonableness, and publicity. After having critiqued the EU Digital Services Act’s approach toward regulating the function recommenders hold, the right to constructive optimization is concretized as an alternative normative benchmark and used as an interpretative lens to enrich ongoing legal initiatives.

## Introduction

Increasingly, offline and online digital environments are streamlined by a diverse set of socio-technical systems that rank, filter, and select the options and choices people have access to: They offer people recommendations, ranging from who they should watch and listen to, the news they should read, the jobs they should apply for, and the commercials they should receive. These so-called recommender systems have become integral to the digital society’s basic structure and are active co-mediators of people’s social and economic affordances and, hence, their enjoyment of fundamental rights, freedoms, and interests (Gabriel, 2022; Naudts, 2024).

Focusing on the deployment of recommender systems on very large online platforms (hereafter VLOPs) and social media VLOPs like X, Facebook, and YouTube in particular, this paper substantiates the legitimate claim consumers, as citizens, have to co-construct their digital environment under conditions of democratic inclusivity. Indeed, due to the public function recommenders exercise in such settings, we contend that EU policymakers should promote governance standards that foster a meaningful reflection of people’s needs, concerns, and perspectives across the content policies and optimization strategies that inform these systems’ functioning. To strengthen citizen-consumers’ political agency doing so, we introduce a “right to constructive optimization” that amplifies people’s fundamental interests within and counters VLOPs’ hegemony over the digital public sphere.^1^

On the personal level, recommender systems can provide genuine utility through filtering content that is more catered to the needs and desires of the users (see among others Danaher, 2022; Monzer et al., 2020). On a societal and democratic level, recommender systems can be designed to push more diverse content and raise the political voice of marginalized social groups (Helberger, 2019; Vrijenhoek et al., 2021). Their potential can also be used in the opposite direction. Research shows how individuals can become the subject of misdirection, deception, and manipulation (Sax, 2021), and social groups of (political) silencing (D’Ignazio & Klein, 2020; Noble, 2018; Brown & Younes, 2023).

Recommender systems’ influence has not blindsided European lawmakers. In recent years, the European Commission opened formal proceedings against X and Meta for their alleged breach of the Digital Services Act’s (hereafter, the DSA) provisions on deceptive advertisements and misinformation (European Commission 2023, 2024b). At the same time, Big Tech leaders, like X’s Elon Musk, have used their voice and platform to weigh on the EU political landscape, including its approach toward tech regulation (Haeck, 2025; Ross & Nöstlinger, 2025). Moreover, as the USA’s shift toward a tech oligarchy illustrates, there can be a deep interconnection between platforms’ pursuit of self-serving economic interests and the geopolitical ambitions of a specific state (Papaevangelou, 2025; Rolf & Schindler, 2023).

Beyond enforcing existing legislation, however, European institutions have re-expressed their intention to further enhance citizen and consumer protection in navigating the digital sphere. The 2022 “Digital Decade Policy Programme” positioned human-centricity, freedom of choice, a fair digital environment, safety and security, solidarity and inclusion, participation in the digital public space, and sustainability as regulatory focal points (European Parliament, Council and Commission 2022). “Digital Fitness Check” initiated by the Commission aimed to assess the resilience of the existing consumer *acquis* in light of technological advancements (European Commission, 2024c). In 2023, the European Parliament issued a resolution questioning the “addictive” design of online services and “the mental health effects of interaction-basedthat (very large) online platforms (hereaf recommender systems (European Parliament, 2023).”

More recently, values like digital fairness, equality, and inclusivity have also been highlighted by President von der Leyen in her Mission Letters to the new Commissioner-Designates at the beginning of her 2024–2029 term. In her Mission Letter to Michael McGrath (Democracy, Justice, and the Rule of Law), she encouraged the recently appointed Commissioner-Designate to start developing a Digital Fairness Act, which should protect people against unethical commercial tactics “related to dark patterns, marketing by social media influencers, the addictive design of digital products and online profiling, especially when consumer vulnerabilities are exploited for commercial practices” (Von der Leyen, 2024a). In her mission letter to Hadja Lahbib (Equality), von der Leyen emphasized the importance of “equality for all and equality in all of its senses (Von der Leyen, 2024b).” As research demonstrates how technological innovations tend to reinforce, rather than replicate, structural inequalities and histories of disadvantage (Eubanks, 2018; Noble, 2018; O’Neil, 2016) and, therefore, amplify the unequal enjoyment of fundamental rights across social groups, these ambitions should not remain mere lip service.

These developments underscore the rightful fear that (very large) online platforms (hereafter VLOPs)—even in the face of existing regulation—are more likely to pursue self-serving optimization logic rather than address the harms their recommendation technologies might reinforce. However, they also demonstrate there is a political willingness to go beyond current provisions to support the protection of citizens navigating an increasingly intricate digital society.

### Research Question, Goal, and Structure

The core question we address in this paper is as follows: How can regulators further promote people’s effective enjoyment of fundamental rights and civic values in digital settings, and how can we make more concrete what these legitimate rights of users are?

This paper motivates a series of foundational values and conditions for an alternative, more inclusive, and democratic approach toward VLOP governance. Rather than viewing the asymmetries in negotiating power exerted by VLOPs as a *fait accompli*, our model empowers citizens and societal interest groups to exercise meaningful choice and voice over, that is, helps regain their political and legal capacity to co-construct, the underpinning logics and imperatives that mediate the digital public sphere. This paper examines the circumstances under which such a “right to constructive optimization” could emerge. In addition, by exploring the relationship between social values and the DSA’s VLOP and recommender provisions, we explore this right’s contribution to ongoing regulatory efforts.

We first strengthen our claim that VLOPs, and the recommenders they employ, perform a public function, that is, to affect people’s access to social justice and fundamental rights. We explain how these functions are not performed in a vacuum but emerge from a dynamic interplay between various societal and technological factors, such as recommender systems and the optimization logics that affect their functioning (Repositioning VLOPs and Recommender Systems). Second, given this public function, we identify a series of values we want people to be able to benefit from within their digital environment: values that should be promoted, rather than undermined, following the deployment of recommender systems on VLOPs (Constructing What? Self-Development and Self-Determination). In particular, we draw from the work of Iris Marion Young and highlight the values of self-development and self-determination as a polestar for future regulatory initiatives (Young, 1990, 2002). In these values, we find a foundational normative basis that captures the socio-technical dynamics VLOPs exert and the degree of social transformation needed to ensure equal access and enjoyment of fundamental rights. Yet, to realize the latter ambition, people should partake in the governance of VLOPs under conditions of inclusivity, political equality, reasonableness, and publicity. Finally, from these values and the corresponding democratic conditions, we concretize our right to constructive optimization and its interpretative relationship with the DSA (Articulating a Right to Constructive Optimization).

This paper expresses the normative building blocks for an alternative, more inclusive digital eco-system. To accomplish this vision, work cannot end there. For instance, questions concerning the enforcement of these idealized standards are critical points of contention, but they remain outside the scope of this particular contribution. Above all, by formulating the contours of a right to constructive optimization, we call upon legislators to be more concrete and ambitious in their democratic vision of platform governance initiatives.

## Repositioning Very Large Online Platforms and Recommender Systems: from Proprietary Market Makers to Socio-technical Information Infrastructures

To render optimization systems more socially responsive, we must reconfigure our understanding and representation of them. In this section, we substantiate our claim that approaching recommenders on VLOPs as proprietary technology at the complete discretion of the platform is too narrow a view and one that ignores the fact that by determining what people see and, perhaps more importantly, what they do not see, VLOP recommender systems mediate the public information infrastructure and, as such, are integral to public interests and civic rights. Secondly, to develop meaningful policies to advance public interests and civic rights in VLOP recommenders, we must broaden our understanding of what we mean when discussing recommenders. Recommenders are not governed by decontextualized, singular, and algorithmic decision-making instances caught within a black box whose innards we cannot comprehend and control. Instead, they emerge through the complex interplay between social and technological processes.

### The Public/Proprietary Function of Recommender Systems on Very Large Online Platforms

Recommendation algorithms are socio-technical approaches to a pressing problem: how to navigate the abundance of information online and decide how to invest attention as a limited resource in a valuable, safe, and relevant way. Recommendation algorithms can help users find music, services, job opportunities, news, videos, or social media posts, depending on the context in which they are implemented, and users can perceive recommendation algorithms as a beneficial and even necessary means to find relevant content in the available time they have (Danaher, 2022; Monzer et al., 2020). Recommenders are useful tools to help consumers find content they like. Most importantly, however, they are also powerful marketing tools for social media platforms (Hendawi et al., 2023).

As “attention management tools,” social media recommenders have a critical function in matching the right people with the right content. What counts as “right” in this context depends on the optimization goals a social media platform pursues. Optimization can be understood as an organizing principle where specific preferred values and/or outcomes are embraced as the most relevant (e.g., user engagement) and where the entire system is—via feedback loops and constant dynamic readjustments—oriented toward the continuous optimal pursuit of the chosen values and outcomes. Optimization logics thus have a tendency to *permeate* systems, determining key decisions at the design, operational, marketing, and even hiring levels (Kulynych et al., 2020). Recommender systems used by VLOPs too can be understood from this optimization perspective. In line with the commercial nature of most social media platforms, the values for which systems are optimized are often commercially oriented: increasing engagement, spending more time with the platform, and bringing profitable content and behavioural advertisements to consumers’ attention (Cobbe & Singh, 2019). They can also achieve the opposite and decrease the visibility of content that might disturb, shock, or offend advertisers (Leerssen, 2020). Powered by troves of personal user information, social media recommendation algorithms can use the knowledge about what groups of users or even individual users find interesting, worthwhile, or relevant to predict, persuade, and increase profits.

As such, social media recommenders are the backbone of the commercial strategy of platforms and valuable commercial assets. Gillespie explains: “A platform is a broker, profiting by bringing together sellers and buyers, producers and audiences, or those in charge of tasks and those with the necessary skills to accomplish them” (Gillespie, 2018, p. 21). Next to being one of the central mechanisms to manage information flows and optimize for revenue, recommender algorithms are also important factors to position platforms in the overall information economy and define the conditions for competition on and between platforms. As proprietary gateways for access to audiences, data, technology, and content, powerful platforms can make and break competitors and determine the conditions of access to markets. Because of their economic and competitive importance, VLOP recommenders are protected by many layers of secrecy (Bodó et al., 2019). This impenetrability is further enhanced by the imaginary of the “black box” that is so popular in describing recommender algorithms as an objective of regulatory attention.

Next to their private, commercial function as “market makers,” the recommendation algorithms of (large) social media organizations also have an increasingly important public function. In their book *The Platform Society*, Van Dijk, Poell, and de Waal signalled already early on that “platforms have penetrated the heart of societies – affecting institutions, economic transactions, and social and cultural practices – hence forcing governments and states to adjust their legal and democratic structures” (van Dijck et al., 2018, p. 2). An important element of this public function of platforms is what the authors call the “platform ecosystem” and “infrastructural core” of the digital society. Recommendation algorithms have a central place in that information ecosystem. This centrality recommenders owe due to their power to determine who sees what and whose content is not seen and how they are instrumentalized to build privately controlled public spheres. From this public function perspective, recommenders are not simply tools that help us find information or tools for platforms to target us with advertising. Instead, the combined recommendation algorithms of social media platforms form the digital information infrastructure over which content and information flow and reach us. This can be information about friends, leisure activities, and job opportunities, as well as information about public events, media content, political communication, and announcements from our governments. There is ample empirical evidence of how recommendation algorithms are designed to influence our exposure to information, our right to being heard, the kind of political messages we receive, and our ability to participate in democratic processes (Haim et al., 2018; Kitchens et al., 2020; Nielsen et al., 2022).

As such, VLOP recommenders not only gate-keep people’s access to and enjoyment of their democratic freedoms and fundamental rights. They also have a substantial public function (Rahman, 2017; Wagner, 2018). Moreover, due to this public function, their optimization should not be a matter for platforms to decide, even if these are proprietary systems and part of the commercial business strategy of a platform. Instead, we argue that VLOPs have a public duty to develop and implement its recommendation algorithms with the interests of citizens and society in mind. As evident as this may sound, as we will show later, this is a step and shift in mindset that the EU regulatory framework, and the DSA’s recommender provisions in particular, still have to take.

### Static Versus Socio-technical: Understanding the Relationality of Digital Ecosystems

Policy debates often tend to concentrate on a narrow, technical understanding and approach that positions recommenders as algorithmic black boxes under the control of a platform. The DSA, for example, defines a recommender system as a “fully or partially automated system used by an online platform to suggest in its online interface specific information to recipients of the service or prioritize that information, including as a result of a search initiated by the recipient of the service or otherwise determining the relative order or prominence of information displayed” (Article 3 (s) DSA). In reality, recommendation systems are not single pieces of software, nor should they be viewed as singular decision-making instances: They are socio-technical instruments that draw from, inform, and push against their social surroundings (Leerssen, 2023; Rieder & Hofmann, 2020).

Algorithms actively co-mediate their surroundings and help establish the conditions under which people face societal harm and injustice, like (historic) prejudice and discrimination. This societal “push and pull” recommenders exert—which adds to their public function—stems from the dynamic interplay between various societal and technological factors (Naudts, 2024). For example, ample evidence demonstrates how data reflect historical prejudice, such as racism and sexism. Unsurprisingly, without intervention, the systems trained on said data will reflect these societal biases (Friedman & Nissenbaum, 1996). However, the reach and impact of stereotyped data are often heightened in automated settings due to the scale at which software is rolled out. Consequently, recommenders further institutionalize a social problem. Even more so, technology does not simply replicate but tends to reinforce and exacerbate existing prejudice. However, algorithms might offer new insights regarding the structuring of society. The provision of recommendations typically depends upon the classification and categorization of people on an aggregate level (Viljoen, 2021). Whereas traditionally, people might have been segmented into categories alongside salient characteristics, like gender and ethnicity, inferential analytics can highlight the importance of less-tangible traits, such as people’s actions, behaviours, preferences, or other monitorable characteristics (Naudts, 2023; Wachter, 2023). As these categorizations determine who and what people see and hear (and vice versa), they have consequences for a diverse set of individuals, groups of individuals, and society at large (Naudts, 2024).

Given these social dynamics, VLOPs cannot be designated as the sole party responsible for creating a better world. If they are to perform a democratic function, they rely on others to succeed in their mission. We may still decide that providers of VLOPs are more responsible for issuing societal change. However, insofar as we want to transform the status quo, we cannot escape the reality that doing so constitutes a shared responsibility (Young, 2011). At the same time, we cannot view VLOP providers, nor the technologies they employ, as monolithic entities. As we argued earlier, VLOPs and their recommender systems are permeated by optimization logics. Recommender systems are informed by decisions on the managerial level, like content moderation policies and practices. Those managerial decisions, in turn, might be informed by advertising and marketing strategies. Data engineers and design staff translate those practices into code, affecting automated content moderation technologies and the information recommender systems display to users. In other words, a whole range of decision-making points and hidden power structures—which embody the optimization logic orienting the systems and organization toward, for instance, user engagement and market share—inform the functioning of recommenders.

In sum, the functions recommenders perform are informed by technical affordances, internal and external value-laden decision-making procedures, corporate and organizational cultures, and social structures. This further strengthens our claim that algorithms should not be seen as black box units but, as we propose elsewhere, and in line with other research (Bratton, 2016; The Ada Lovelace Institute, 2021), as a complicated stack of socio-technical operations that each offers their point for regulatory intervention (Naudts et al., 2024b).

## Constructing What? Self-Development and Self-Determination

Given recommenders’ public function, the critical question becomes: What public values should VLOPs promote to protect, or even enhance, people’s access to effective freedoms and fundamental rights?

In the following section, we draw from Iris Marion Young’s politics of difference to propose self-development and self-determination as two fundamental and interrelated underlying values every person should be able to enjoy. In the first step, we clarify both values in relation to recommenders. The value of self-development refers to people’s ability to “learn and use satisfying and expansive skills in socially recognized settings, and enable them to play and communicate with others or express their feelings and perspectives on social life in contexts where others can listen” (Young, 1990, 2002, pp. 31–32). The value of self-determination concerns people’s ability “to participate in determining one’s action and the condition of one’s action” (Young, 2002, p. 32). As such, these notions overlap with the European Union’s commitments to human dignity, freedom and autonomy, equality and solidarity, and its ambition to ensure the effective enjoyment of fundamental rights. Rather than sharply defined legal concepts, however, both values are best understood as a normative prism through which the aforementioned EU commitments, including their implementation in the law, as well as platforms’ optimization logics, can be critiqued and readjusted. Second, to realize both values, people’s digital environments should be governed by institutional standards based on inclusion, political equality, reasonableness, and publicity. Together, we argue that these two values capture many of the concerns that characterize the digital society: power asymmetries, structural inequalities, unfairness, lack of choice and agency, deceptive and addictive design strategies that govern the relationship between users and VLOP recommenders, and people’s freedoms and opportunities being governed by commercial logics. At the same time, these values give substance to a more constructive role of recommender systems for users. Finally, we highlight how both notions incorporate societal and collective dimensions mandating a regulatory turn that supersedes individualism.

### Self-Development and Recommenders

The value of self-development concerns the conditions for people to learn new capacities and express their experiences as social equals (Young, 1990, p. 37). As clarified by Young, the value of self-development reflects the normative ambitions of the capability approach (Nussbaum, 2011; Sen, 1979, 2009; Young, 2002, p.31). The capability approach is a normative and theoretical framework to enhance the substantial freedoms or opportunities people have to realize alternative beings and doings (Nussbaum, 2011; Robeyns, 2016; Sen, 1979). According to Robeyns, capabilities “denote the freedoms that have been cleared of any potential obstacles, in contrast to mere formal rights and freedoms” (Robeyns, 2016). Importantly, people only have a real chance to pursue valuable life goals, that is, *substantive* freedoms, when they also have access to the appropriate emotional, psychological, social, institutional, and material support needed to realize them (Allen, 2008; Fraser, 1995; Young, 1990).

In this particular context, socio-technical systems act as an external (environmental) condition that can either limit or enhance people’s capabilities: As recommenders increasingly govern the interpretation and navigation of the digital society, they influence choices people make in the skills and capacities they want to develop, how they will be created, the experiences they gain from them, including their communication to others, etc. Social media recommenders might up-rank certain music visible on social media platforms, influencing what genres become popular and which become niche. Those choices impact the people active in the entertainment industry and the freedoms they enjoy. At the same time, they can also impact the freedoms listeners want to realize, including which concerts to go to or instruments to play. Recommenders that push medical misinformation during a pandemic affect people’s effective freedom to maintain their bodily health and integrity. Politically speaking, Palestinians have the mental and physical capacity to develop and persuade others of their plight through political reasoning. Human Rights Watch and Access Now reported how Meta’s policies and practices have been silencing voices in support of Palestine and Palestinian human rights on Instagram and Facebook (Brown & Younes, 2023; Fatafta, 2024). The constraints imposed by VLOPs, and instrumentalized by content moderation and recommender systems, prevent Palestinians from converting this capacity into the desired freedom to participate as equals in discussions regarding their self-governance as a people. In this example, recommender systems limit the capacity to develop (political) self-determination, highlighting the interdependency and reciprocal relationship between both values. Indeed, this limitation on the capacity of self-determination further constrains Palestinians’ definition and pursuit of other wants and needs they wish to develop. As the digital society operates on structures of injustice, similar barriers are faced by other cultural minorities or historically disadvantaged groups. On the positive side of the spectrum, however, recommenders could help people learn, develop, encounter, and express new experiences. For example, we could assess whether VLOPs should actively promote the integration of diversity metrics if doing so would enhance people’s overall ability to act as democratic citizens, where people’s exposure to the lived realities of others raises their overall knowledge of other people’s needs, perhaps also their ability to engage more emphatically (Vallor, 2010). In the case of a global pandemic, we could evaluate whether online platforms should promote scientific information to citizens so they maintain their bodily health and integrity.

Of course, platforms and recommenders do not operate in a social vacuum. And while they certainly are not the only external obstacles people might face in realizing their freedoms, it would still be unwise to consider their impact negligible. Once we know under what conditions recommenders threaten to impact people’s self-development negatively, we can start to explore whether platform operators carry a positive duty to restore, maintain, or perhaps even enhance human capabilities, and if so, how, when, what for, and through which means (Coeckelbergh, 2010, 2011). The EU’s fundamental rights could be seen as substantive freedoms each person needs to lead a dignified and autonomous life. Martha Nussbaum, one of the founding figures of the capability approach, nonetheless sees significant differences between capabilities and the traditional fundamental rights paradigm (Nussbaum, 2006, pp. 284–288, 2011). For one, capabilities impose a positive duty to transform entrenched and structural inequality resulting from discrimination and marginalization. Second, capabilities challenge the conceptual independence between the civil and political (the public) and the social and economic (the private). As such, the value of self-development endorses a view where VLOP providers should proactively promote the full enjoyment of fundamental rights rather than limit their potential out of financial interest. Of course, in so doing, VLOP operators must not forego of their fundamental freedom to conduct a business, as enshrined in Article 16 Charter of Fundamental Rights of the European Union. Yet, to ensure the balance and sustainability of the digital public sphere, other fundamental interests should be given the necessary space to meaningfully enter the conversation.

### Self-Determination and Recommenders

The value of self-determination gives shape to the notion of autonomy. VLOPs mediate people’s options, choices, and life paths. If so, citizens might only become autonomous actors in pursuing and enjoying these freedoms when they are also given effective agency over the choices and conditions that govern these digital ecosystems. This political control should extend to the functions technology performs as their deployment affects the mental, physical, social, and material resources people need access to exercise choice and control over the conditions that govern their lives.

Generally speaking, recommender systems might improve people’s ability to exercise agency over their everyday choices. As Danaher notes, recommender systems can be agency-enhancing as they help “identify and select among options that might (or might not) be conducive to [their] goals” because they “filter choices and reduce the feeling of being overwhelmed” (Danaher, 2022, p. 268). Yet, without appropriate governance mechanisms, recommender systems can turn the online environment into a space of arbitrary or unchecked control (Gräf, 2017; Pettit, 2012). Following Pettit, Young argues that an interference is arbitrary “when it is chosen or rejected without consideration of the interests or opinions of those affected” (Young, 2002, pp. 258–259). VLOPs’ current position could be described as one facilitating algorithmic forms of domination (Danaher, 2022; Gräf, 2017; Pettit, 1999, 2012). Citizens have little to no control over how recommenders are deployed, both in terms of their purpose and their functioning. Recommender systems limit, replace, or create the options, content, and goods people see and access, drawing from citizens’ data, behaviour, or other monitorable actions, and do so under conditions primarily determined by operators and according to their preferences. Addictive and deceptive design strategies, including dark patterns, allow VLOP providers to manipulate people into making choices they would otherwise not make or that best favour the interests of providers. Recognizing these risks, the European Parliament therefore proposed introducing a “digital right not be disturbed,” which could be viewed as one way to operationalize self-determination. Such a right would “empower consumers by turning all attention-seeking features off by design and allowing users to choose to activate these features by simple and easily accessible means, possibly with an attached mandatory warning of the potential dangers of activating these opt-in features, offering consumers real choice and autonomy without burdening them with an information overload” (European Parliament, 2023).

Importantly, however, people’s self-determination is curbed as soon as the ability to interfere arbitrarily exists, not only when this interference is actively exercised. This potential to interfere is especially prescient given the power asymmetries citizen-consumers face. From a knowledge perspective, design choices are kept hidden or only vaguely described. And even if sufficiently granular information is provided, actual insights might only be derived by those with sufficient technological expertise. This informational opacity can also obscure subsequent changes to the recommender model. Given the resources needed to maintain and comprehend the infrastructures and software on which platforms and recommenders operate, the risk of arbitrary interference might never arise. Consequently, lawmakers should strive to limit the ability of actors, whether the providers themselves or political and economic stakeholders that can exercise control over VLOPs, to abuse their position of power.

One challenge recommenders do pose is that their agency-enhancing functions inevitably rely on choice limitations themselves. Recommenders always alter the options people have access to in some specific way. Thus, questions concerning the impact of recommenders on self-determination are (at least) twofold. We should identify those “areas of choice” or substantive freedoms recommenders can and cannot justifiably interfere with. For instance, should platforms be allowed to provide political recommendations? Once we decide recommenders can interfere with specific options and choices, we should identify under what conditions they can rightfully do so. As mentioned above, however, self-determination also mandates for people to have an effective opportunity to exercise political agency over these two questions. And to offer people this agency, we also need to define a series of principles that can make the governance of recommenders more inclusive.

### The Need for an Inclusive Governance of VLOPs

A democratic decision, Young argues, is “normatively legitimate only if all those affected by it are included in the process of discussion and decision-making” on “equal terms,” that is, offer those affected an “equal right and effective opportunity to express their interests and concerns”(Young, 2002, p. 23). Young notes how inclusion “embodies a norm of moral respect: persons (and perhaps other creatures) are being treated as means if they are expected to abide by rules or adjust their actions according to decisions from where determination their voice and interests have been excluded” (Young, 2002, p. 23). Given the decisions that govern VLOPs affect people’s access to self-determination and self-development, regulatory efforts should make the digital environment more favourable, inclusive, and participatory and include the interests of citizens, social groups, and society. To realize this model of inclusive democracy, we need political equality among interest groups, a willingness to listen to the experience of others, and an environment of public accountability.

Regulators could enhance and broaden citizens’ resources to exercise (deliberate) control over recommender systems (See also Gräf, 2017). And, where consumers agree to have their choices curated by others, they should have access to intelligible information to understand the rules of engagement, the conditions under which curation occurs, and the envisaged consequences thereof. Likewise, people’s participation in the ideation and design of recommender systems should be more actively encouraged. In this context, the principle of reasonableness adds to participation an active commitment for those involved and actors with power and privilege to listen to the needs, concerns, lived realities, and experiences of those their technologies might affect. In this context, reasonableness “must not be understood as implicating some thin and disembodied ideal of rationality but instead constitutes a willingness for openness: open to listen, show respect for, understand, and be persuaded by Others, even when their position is communicated disorderly or through emotion, anger, hurt or passion and to look beyond economic and majoritarian interests” (Naudts, 2024, p. 708; Young, 2011). And though doing so ex ante might not always be feasible, regulators should mandate publicity to allow people or their representatives an opportunity to contest governance decisions that threaten to impact or restrict their right to self-development and self-determination unduly. For example, when VLOPs claim their systems are fair, we should be allowed to scrutinize their vagueness: What do they mean, and what normative assumptions and implications result from their use of the notion? Importantly, the demand to listen to others also means that those others should be able to impact and change decisions that were already made and that reasons can be given as to why certain viewpoints could not be considered.

Undoubtedly, when governance procedures promote publicity and contestation, tension and opposition will surface. This “agonism” should instead be seen as an opportunity to further inform our views on recommenders (Appelman, 2025; Sax, 2022). Yet, digital inequalities and power asymmetries unduly limit the emergence of competing narratives. Where a few actors dominate the digital environment, people’s ability to choose a different service provider offers a different set of governing conditions, reducing the capacity to exercise choice and control. One could even argue that it is precisely the lack of meaningful alternatives, and the grasp certain VLOPs hold over the digital public sphere, from which the need to democratize these platforms emerges. Public institutions and regulators thus hold a crucial role. By organizing spaces of contestation and resourcing underrepresented interest groups in their ability to push back against dominant narratives, regulators can help transform existing business practices or enable meaningful alternatives to arise. At the same time, competing narratives might emerge as alternative digital spaces come about.

### Shared Goals: Self-Development and Self-Determination as Social and Egalitarian Values

Self-development and self-determination are not individual values. Whereas individuals might ultimately enjoy the benefits of these values, they embody the egalitarian ideal that their realization should be available to all, which is a shared social endeavour reliant on both individual and collective efforts.

First, following a critical perspective, we can identify various forms of structural inequalities within contemporary societies. Certain social groups face historical disadvantages, and recommender systems might mirror and reinforce the stereotypes they face. For example, a news recommender system might only push content that depicts a particular cultural minority as violent or criminal; positive stories about this group remain hidden. The recommender can act as a technological barrier that limits people’s cultural recognition (self-development), undermining their capacity to meaningfully exercise choice over the conditions that govern their lives (self-determination). As mentioned above, however, we cannot rely on individual or isolated interventions to address this situation. As a news reader, you might be able to opt out of that platform, but that does little to address the problem at its core. Likewise, recommenders might only promote meaningful political diversity when they access sufficiently diverse political content. In other words, we must implement concerted and structural measures to secure people’s enjoyment of both social values. Moreover, in societies characterized by significant social and economic inequality, we must acknowledge that more resources—whether they be cultural, social, economic, procedural, or institutional—must be allocated to correct and dismantle the structural barriers some groups face in their access to these values. In this sense, to realize equality, some form of inequality must be introduced.

Second, by being cognisant of these individual and collective dimensions, we can also better capture the group-level functionings of technology. Technologies often operate on aggregated or population level, rather than individualized insights (Viljoen, 2021). When a person A is recommended item X, because the system knows people similar to A also liked X, it merely conveys information about individuals as group members, rather than individuals as individuals (Vedder, 1999; Viljoen, 2021). Consequently, the type of control recommender systems’ exercise often takes place on the collective level first before ultimately benefitting or harming individuals as members of these collectives. Hence, individual self-development and self-determination and the protection of group interests cannot be achieved through individual or local measures alone but must incorporate more collective-oriented sensitivities.

Third, this form of egalitarianism also asks of every person—and not only platform operators—a willingness to consider the needs and experiences of others. This ultimately renders social justice, or the transformation of the status quo, a shared commitment. Of course, this need not imply that privilege and power do not define the level of responsibility each entity or individual carries (Kasirzadeh, 2022; Young, 2011). Given the influence VLOPs carry, one can rightfully assign to them additional duties of social care. Attempts to accommodate both individual and collective-level needs are likely one area in which tensions regarding the governance of recommenders will arise. Yet, as mentioned, incorporating some form of agonistic pluralism might be productive. The point is for governance standards to foster and bring these tensions to the forefront and give them space rather than preclude them altogether.

## Articulating a Right to Constructive Optimization

Given the public and private functions recommenders perform, we argue for a critical adjustment of recommender systems’ governance. Business interests certainly matter—the freedom to conduct a business is protected under the Charter after all. Still, their pursuit should be aligned with, rather than trump, other fundamental rights and democratic values.

In this section, we present a tentative step toward formulating a right to constructive optimization that protects the values of self-development and self-determination under inclusivity, political equality, reasonableness, and publicity conditions. Drawing from our normative analysis thus far, we first propose a series of guiding principles that can help evaluate whether citizens can actively participate in the governance of socio-technical ecosystems in ways that are seen and heard. In the second phase, we use these principles to evaluate the DSA’s proprietary approach to recommender systems. In the third phase, and drawing from our normative analysis and evaluation, we formulate what a right to constructive optimization could look like. Finally, we use this renewed right as an interpretative lens to inform and improve upon the DSA’s recommender provisions. In so doing, this section *translates* the values of self-development and self-determination into an actionable, non-exhaustive, series of substantive guidance through which existing platform governance strategies can be assessed and operationalized.

### Guiding Principles

VLOPs, from the cradle to the grave, should promote self-determination and self-development under conditions of inclusivity, political equality, reasonableness, and publicity. Following these ideals, we propose a series of governance principles through which regulative efforts can be examined on their ability to assure for citizens, whether as individuals or as members of (social) groups, equal enjoyment of self-development and self-determination. In the brackets, we highlight to which political ideal(s) each evaluative standard roughly corresponds:

- Have an actual and actionable say in the optimization goals pursued within digital ecosystems (**Inclusivity and Political Equality**).
- Exercise meaningful choice and voice, which requires the presence of alternative options, both in relation to a particular recommender system’s functioning and in relation to other operators, including service providers and platforms (**Political Equality and Reasonableness**).
- Are included, represented, and have one’s voice heard during the ideation, design, deployment, and evaluation of recommender systems, meaning they should have access to (collective) participation and contestation mechanisms from the cradle to the grave (**Inclusivity, Political Equality, Reasonableness, and Publicity**).
- Understand the rules of engagement, including how systems function, the normative assumptions upon which they have been built, for which purposes they have been optimized, and the consequences such optimization strategies entail on the content users see (**Publicity**).

To truly ensure these conditions can be enjoyed by all, in governance and effect **(Inclusivity and Political Equality)**, particular attention must be paid to marginalized or otherwise vulnerable communities. If not, the digital environment will become an additional barrier to breaking down in the fight against the structural inequalities and injustice they already face. In this context, constructive optimization also pertains to the interests of those who may not directly interact with a recommender or are (structurally) underrepresented and excluded because recommendation algorithms are not only a tool for users to discover information but also a means for non-users and other parties affected by the algorithmically mediated choices to be discovered (See also: Napoli and Sybblis, 2007). For instance, certain (social) groups might not have access to or interact with digital technologies, but that does not mean their interests on these spaces should not be represented or have some form of representation (Naudts, 2024).

### The DSA’s Proprietary Market Approach Toward Recommenders

Within the EU’s Digital Acquis, the DSA is the regulatory instrument that governs the use of recommender systems on VLOPs. Through the lens of the overarching principles defined in the last section, which reflect the governance conditions under which equal self-development and self-determination can be realized, this section evaluates several key areas within the DSA that govern the use of recommendation algorithms on platforms: (a) recommender system transparency and agency (Articles 27 and 38 DSA), (b) the prohibition of dark patterns (Article 25 DSA), and (c) the envisaged (societal) consequences platform operators must consider when deploying recommendation systems (Articles 34 and 35 DSA). Finally, we critique a significant omission within the DSA’s framework: a lack of substantial data governance standards.

#### Recommendation System Transparency and Choice

At the core of the DSA’s recommender provisions is Art. 27 DSA. Article 27 DSA applies to all platforms, including but not limited to VLOPs. According to Art. 27 DSA, “providers of online platforms that use recommender systems shall set out in their terms and conditions, in plain and intelligible language, the main parameters used in their recommender systems, as well as any options for the recipient of the service to modify or influence those main parameters” (Art. 27, para 1). Those “main parameters” should explain to recipients of recommenders why certain information has been suggested (Art. 27, para 2). The DSA further specifies that those parameters should at least comprise the criteria most significant in determining the information offered and the reasons for their relative importance.

Aimed primarily at providing recommender transparency, in practice, Article 27’s recommender obligations do too little to inform and help users understand *platforms’ rules of engagement*. First, the DSA’s reliance on parameters to offer users insight concerning their navigation of platforms appears misguided. Though no definition is provided under the DSA, the consumer Omnibus Directive (OD) notes that parameters are “any general criteria, processes, specific signals incorporated into algorithms or other adjustment or demotion mechanisms used in connection with the ranking” (recital 22 OD). These parameters, however, differ in their comprehensibility and explicability. Whereas certain criteria are more or less humanly comprehensible (e.g., geographic information or preferences), others, like telemetry data, clicks, and device touches, are more abstract and difficult to parse (Edwards & Veale, 2017; Wachter, 2023). In the case providers do succeed against the odds in offering comprehensible information regarding the parameters that were used, users might still lack direct insight into the optimization tactics, normative aims, and social consequences these choices represent for platform governance. Users might understand that their clicks represent their engagement, but not necessarily that systems designed to ruthlessly optimize engagement can also have emergent societal consequences, such as excluding minority groups or views from their digital perimeter. Second, the requirement for information only to be present in the terms and conditions is a criterium that has been repeatedly criticized because of the unlikelihood that consumers will actually read the terms of use (Obar, 2015).

Even if we assume the rules of recommender engagement are meaningfully conveyed, users would still lack the opportunity to exercise *meaningful choice and voice*. Beyond the obligation for VLOPs to offer users non-profiling-based recommendation options (Article 38 DSA, see also below), the DSA does not mandate providers to give recipients *alternative recommendations*. The DSA leaves it at the discretion of platforms to actually give users a choice between different recommendation logics. Art. 27 DSA only stipulates that in the event a platform should decide to do so, this must be done in a user-friendly way so that users can easily access, modify, and select their preferred options from the VLOPs’ interface (Article 27, para 3). Put differently, Article 27 DSA does not enable citizens to alter or influence the parameters and weights themselves, or contest the choices of parameters made by the platform. Instead, it is only concerned with the form in which such options are offered, if at all.

Despite their public function, the DSA approaches VLOP recommender systems from the perspective of a proprietary technology whose design is at the exclusive discretion of the platform, within the boundaries of the law. Effectively, Article 27 DSA does little to address underlying structural asymmetries. Neither does it increase the power of users to exercise voice and express their preferences for a particular recommendation logic, nor does it offer users with a right to claim a real choice. In sum, Article 27 DSA does not offer citizens, either as individuals or through representative organizations, an *actual and actionable say* over the ideation, design, and deployment of the recommendation systems that mediate their online surroundings. Consequently, users also have no (situated) control or contestation mechanisms to influence choices made in the digital public sphere, and the downstream impact the former might haveon their overall effective freedoms either.

Perhaps the most promising agency-enhancing tool regarding the deployment of recommender systems is Art. 38 DSA. This provision exclusively applies to VLOPs and stipulates that VLOPs “shall provide at least one option for each of their recommender systems which is not based on profiling,” and this choice should be accessible from the online interface where recommendations are presented (Article 38 DSA, read together with Article 27 DSA). Art. 38 DSA is relevant for the realization of the right to self-determination in the sense that it must give users a real choice between different recommendation logics—one profiled and a non-profiled one (Starke et al., 2023). This is particularly important as users’ data is also crucial for the profiling of others (see, e.g., Viljoen, 2021). Moreover, this provision will potentially give users a choice to compare how the recommendations look with and without profiling. Users might even study these systems together using this method to gain collective understanding—although significant barriers continue to exist for both individual and community engagement (see, e.g., Mahieu et al., 2018). However, there remain many downsides to this particular provision. First, there is no obligation to make the alternative system that is not based on profiling have any utility for individuals. It is likely that this option will languish, deliberately neglected by the platform to minimize those individuals wishing to choose it, even where a desirable alternative can be envisaged. In other words, the choice on offer is not meaningful to allow competing structures to emerge on platforms. Second, this option to switch off recommendations based on profiling does not automatically prohibit profiling itself for other purposes—this is governed primarily by data protection law, and multiple methods to express agency may be confusing. Switching off any form of optimization may leave individuals unable to navigate complex informational landscapes—a negative protective provision, but without a positive element promoting freedom, self-development, and self-determination. Without genuine alternatives, this two-option framework is easily abused.

#### The Prohibition of Dark Patterns

Though the DSA offers users little actionable choice and voice over recommender systems, the regulation nonetheless recognizes that architectural and technological design choices can reduce people’s autonomy. Article 25 DSA, the so-called dark patterns provision, is particularly interesting in this context. Art. 25 DSA stipulates that “providers of online platforms shall not design, organize or operate their online interfaces in a way that deceives or manipulates the recipients of their service or in a way that otherwise materially distorts or impairs the ability of the recipients of their service to make free and informed decisions.” Dark patterns constitute and facilitate (arbitrary) interferences with people’s choices and, in so doing, can enable platforms to favour certain economic and political narratives over others surreptiously. The DSA offers three examples of such epistemic distortions: (a) presenting certain choices more prominently than others, (b) using pop-ups to interfere with users’ choices, and (c) making the procedure for terminating a service more complex than subscribing to it (Article 25 (3) DSA). By protecting people against deceptive or manipulative tactics, the DSA hopes to ensure user interaction under fair conditions of engagement.

Unfortunately, this provision falls short in its ambitions to curb undue limitations to people’s self-determination. For one, Art. 25 has a limited scope. Art. 25 DSA does not apply to practices covered by the General Data Protection Regulation (GDPR) and the Unfair Commercial Practices Directive (UCPD), limiting de facto the scope of the provision to forms of misleading non-commercial advertising (Art. 25, para 2 DSA). Furthermore, Article 25 DSA merely tackles the presentation of pre-made choices and not the way those choices have been made in the first place. In other words, there is no obligation on platforms to refrain from optimizing their recommender systems to maximize engagement or optimize for content that sells or gets users “hooked” (in the sense of addictive designs), even if doing so conflicts with legitimate information needs and democratic rights of users.

#### Recommendation Systems and Systemic Risk

Whereas the dark patterns provision targets the risks deception and manipulation pose to free and informed decision-making, the DSA’s systemic risk provisions in Art. 34 and 35 DSA intend to address the broader, societal consequences VLOP choice environments effectuate in a more comprehensive manner.

First, on a societal level, providers should “identify, analyse and assess any systemic risks in the Union stemming from the design or functioning of their service and its related systems, including algorithmic systems, or from the use made of their systems” (Article 34 (1) DSA). Systemic risks might threaten citizens and society in a broad sense and include the amplification and wide dissemination of illegal content, foreseeable negative effects on the exercise of fundamental rights or high-level consumer protection, and dangers to civic discourse and electoral processes. Evidently, in differing ways, the aforementioned systemic risks can threaten, on an individual and collective level, directly and indirectly, people’s ability to pursue and communicate the life projects they value (self-development) and the degree of autonomy needed for doing so (self-determination).

There is, however, an inherent tension between the individualized interests of users in a right to self-determination and self-development and the focus on systemic risks as design limitation for platform recommenders. As Broughton Micova and Calef explain, “a risk is therefore systemic when it can lead to harm to individuals at a large scale or to systems essential to the governance and good functioning of society” (Broughton Micova & Calef, 2023, p. 11). In other words, and in line with regulatory choices elsewhere (such as the AI Act), scale matters when determining whether or not recommenders pose a risk, and the focus is on risks to society, not individual users. In contrast, self-determination and self-development are also, though not exclusively, about the individual’s relationship to platforms and how recommender systems shape, influence, and affect the situated reality of individual audience members.

Second, the DSA could be interpreted as to mandate providers to consider the deeper socio-technical layers, dimensions, and interactions that inform the functionalities of VLOPs (Article 34 (2) a–e DSA). In their assessment of risks, providers are among others tasked to reflect on (a) the design of recommendation and content moderation systems; (b) managerial decisions concerning applicable terms and conditions, including their enforcement; (c) broader economic conditions regarding the selection and presentation of advertisements; and (d) the social implications of data-related practices. Though responsibilities vis-a-vis marginalized or otherwise vulnerable communities are recognized, they have unfortunately been relegated to the recitals: “obligations on assessment and mitigation of risks should trigger, on a case-by-case basis, the need for [VLOPs] to assess and, where necessary, adjust the design of their recommender systems, for example by taking measures to prevent or minimize biases that lead to the discrimination of persons in vulnerable situations.”

Correspondingly, mitigation measures aimed to address the identified risks may include the design of the online interface, adapting terms and conditions at the managerial and operational level, adjusting the presentation of advertising, or adapting the design of their recommender systems themselves (Art. 35 (1) DSA). In addition, reference is made to the role of trusted flaggers and other providers of online platforms or online search engines considering that platforms operate not in isolation but that their operations are influenced by the broader information ecology in which they operate. In acknowledging the importance of the organizational and social cultures surrounding VLOPs and recommender systems, the DSA resonates in part with a more stack-oriented approach toward platform governance outlined above.

Having said so, the identification of systemic risks too is left mainly to the platform’s discretion. One of the strengths but also the weaknesses of the DSA’s procedural approach is the lack of substantive guidance on how to interpret and define suitable benchmarks. This leaves the interpretation of when the fundamental rights and freedoms of users are at risk largely to self-assessment by VLOPs as the controllers of a proprietary technology. Moreover, fundamental rights here are not guiding principles for the design of recommenders but a negative condition: under the risked-based approach gives VLOPs free rein to determine to which ends and in which ways to design recommender systems, *as long as those do not create risks for fundamental rights*. The DSA does not introduce a corresponding obligation to design recommenders in a way that either (a) actively involves citizens in constructing the conditions under which VLOPs can affect their digital surroundings or (b) actively encourages VLOPs to enhance people’s freedoms under conditions other than self-serving interests that (c) consider the particular needs and interests of those most vulnerable.

#### Data Obligations for Platforms and Recommenders

One crucial component left partially ungoverned by the DSA is the data upon which recommenders rely. The quality and characteristics of recommender systems are strongly determined by the content pool from which socio-technical systems can draw. As such, data are reflective of the informational narratives that can emerge on platforms, and consequently, how people’s navigation and encounters on those platforms will be constructed. What people see and do not see online, and consequently, the choices and effective freedoms they have within and outside these spaces, are shaped by data. For example, to present diverse recommendations, recommenders must have access to a diverse set of contents, reflecting different perspectives, topics, and genres. Similarly, if a recommendation system was to contribute to the self-development and self-determination of a diverse set of users, it would need to be able to draw on a pool of information that reflects the diversity of informational needs of its users.

The approach that the DSA takes is a different one. There are no quality requirements at the input level as long as the content is lawful. For instance, there are no requirements that the pool of information must not be biased toward particular political views, cater to minorities, or reflect different languages. Imposing such requirements on platforms is also hardly feasible or desirable as most content is user-generated, and platforms cannot or should not be expected to curate input content actively. This highlights the importance of the contestability of moderation decisions and the terms of use and usage policies based on those ﻿decisions﻿ (Appelman et al., 2021), including the ability for minority groups to contest moderation or curation actions that unjustifiably limit the visibility of their content also matters.

### A Right to Constructive Optimization

To the extent that the recommender provisions in the DSA create rights for citizens and consumers, our analysis shows that people’s interests are narrowed down to a take-it-or-leave-it choice. Recommenders are part of an entire social infrastructure package that platforms are free to design and control according to their commercial interests and objectives. The only exceptions are situations in which the decisions of a platform result in systemic risks for fundamental rights and society. Yet, this public dimension of recommendation algorithms is defined as a negative scenario, a situation that limits the commercial discretion of a platform. Users and affected non-users are at the receiving end, with no or very limited say in the configuration of recommenders and, consequently, no room to harness these technologies in ways that suit their self-development and self-determination and their enjoyment of fundamental rights.

To tackle the resulting digital asymmetry between platforms and users (Helberger et al., 2021) and account for the public function of VLOP recommender systems, we proposed a right to constructive optimization elsewhere (Helberger et al., 2024b; Naudts et al., 2024a, b), which we will further specify here and apply as a new analytical lens to inform the interpretation of the DSA. The “constructive optimization” notion captures at least three interrelated regulatory dimensions. First, we wish to give back political power and agency to users as non-users to co-create or co-construct their digital environment. Second, in so doing, we view recommender systems as socio-technical instruments that can help construct, realize, or transform of people’s digital living sphere. Finally, our view is constructive as it acknowledges the market foundations and goals of the EU but believes more can still be done to align these historical roots with the EU’s contemporary fundamental rights objectives (Muir, 2013). In other words, it attempts to incorporate the values of self-development and self-determination under conditions of inclusivity as an integral building block toward constructing our digital environments.

**A right to constructive optimization**.

1. The design, operation, and evaluation of the socio-technical recommendation systems employed by Very Large Online Platforms must be organized in a way that actively promotes people’s enjoyment of self-development and self-determination, either as individuals or as members of social groups—including persons that are marginalized and/or otherwise rendered vulnerable—as to ensure, *inter alia*, the equal protection, realization, and enjoyment of their fundamental rights, including the right to privacy, equality and non-discrimination, and freedom of expression.
2. When organizing the ideation, design, operation, and evaluation of these systems, utmost consideration must therefore be given to the viewpoints of individuals and communities expressed to the platform via easy-to-use communication channels made available for this purpose. Where changes in recommender design, desired by certain individuals and communities, cannot be implemented, such refusal should be accompanied by fair and reasonable, clearly motivated, and publicly communicated reasons that reflect due consideration for the affected people’s interests.
3. Responsible providers of VLOPs must document and make public information on choices made during the ideation, design, and development process of recommendation systems to enable third parties, including affected end-users, citizens, civil society organizations, academia, and the regulator, to assess whether a system is sufficiently aligned with democratic values**.**
4. The burden of proof that this obligation has been complied with is on the platform operator.

The right to constructive optimization goes beyond a risk-based approach in that it establishes a positive obligation to include and consider the interests of users and a diversity of users along the entire development chain, from ideation, design, and deployment to the evaluation of recommender systems, thereby contributing to the principles of inclusivity, political equality, and reasonableness outlined earlier. Importantly, this obligation extends to a commitment to also consider the structural inequalities that characterize our digital society and act as barriers that impede historically disadvantaged groups or otherwise marginalized or vulnerable (social) groups in their enjoyment of self-development and self-determination.

To render these proactive duties substantive, rather than formal, this provision also imposes onto providers the duty to actively and meaningfully *listen* to and engage with the diverse wants and needs of people with a prospect to initiate change. Participatory design methods could constitute a reciprocal engagement with individuals and communities if not treated solely as a transactional exercise but as an enduring process that empowers and rewards affected persons for sharing their lived experiences and perspectives (Birhane et al., 2022; Sloane et al., 2022). However, given people’s diversity in needs and technology’s emergent capabilities, ex ante measures like co-design should always be complemented with strong accountability and contestation measures ex post. Of course, it may not be possible to accommodate all the design changes requested by affected persons or communities. At the same time, the need to run multiple recommender systems in parallel to address people’s differing needs and interests should not, in itself, be a reason to refuse to implement such changes.

Importantly, to realize the right’s ambitions, the notions “recommender” and “recommender design” should not be conceptualized narrowly: For our purposes, these concepts do not target a local, technical algorithmic system but capture more broadly a stack of different operations. Such an approach embraces the insight that beyond the surface interface level, *every* part of the recommender system—from the software, hardware, infrastructure, organizational, design principles, and so on—works in concert to, ultimately, create particular tools, interfaces, and functionalities (Naudts et al., 2024b). Effective recommender governance thus acknowledges that a system’s technological design results from company goals determined by management, business KPIs, hiring practices, data collection and analysis practices, iterative software design philosophies, UX/UI design choices, data training models, and so on. By-design interventions that are focused on the “recommender” as a discrete and local black box, rather than an intricate and interconnected decision-making structure, tend to miss the forest for the trees (see also Kasirzadeh, 2022). Indeed, given the aforementioned social interdependencies, attempts to re-orient a system’s values through technological interventions alone are futile. The stack perspective opposes techno-solutionism of this kind and favours an integrated and structural approach toward platform governance: Beyond technological interventions, corrective measures could also take aim at the stack’s organizational and societal layers. In this respect, the envisaged stack approach also extends upon the by-design approach found in EU data protection law. While the latter does refer to technical and organizational measures decided upon by the controller (See Article 25 (1) GDPR, and European Data Protection Board, 2020), the value-driven proposal we outline adds to these standards of inclusivity that facilitate an active dialogue with those affected by recommenders.

Access to information is a precondition for establishing political dialogue and agency. In this context, forward-facing documentation policies help establish accountability, reasonableness, and transparency on the public level, where insight into managerial and strategic choices can help users better assess and where necessary contest, the logic of a recommender system. Publicity requirements thus cover a wide range of information across the corporate ladder, including the performance of impact assessments, data production standards and processing practices, and automated decision-making strategies. Information obligations should also extend to the actual efforts made by providers to listen and engage with their users and affected non-users in ways that gave choice and voice, that is, allow for meaningful change. At the same time, accountability mechanisms should enable individuals and interest groups, such as civil society and research institutions, to operationalize information, that is, to scrutinize and contest the normative assumptions and choices made by VLOP providers. By making public and contestable the internal and external procedures governing the recommender stack, providers must substantiate their having listened to others and their willingness to engage with new insights if these arise.

Seeing the public function of recommenders, we argue that providers of VLOPs have a heightened responsibility to redress disadvantage and facilitate social change. Hence, we propose that to realize political equality and reasonableness, it is necessary for these actors to also carry the burden of proof of their meeting these standards. Such a reversal also makes sense in light of the considerable information asymmetries and the difficulty or undesirability of giving users access to internal company documents. The reversal of proof thus helps eliminate power imbalances between providers, users, and other interest groups.

Above, we have outlined the right to constructive optimization in broad strokes; it is a normative anchorage point toward a more alternative, more inclusive model for platform governance and recommender design in particular. For one, its implementation in practice depends upon social, economic, and technical structures in which a particular recommender system operates. Once this broader “stack perspective” has been obtained, we can formulate a more concrete set of immediate and actionable measures, some of which we have explored in prior work (Naudts et al, 2024b). Second, further iterations should also include considerations of secondary rule-making and enforcement to mitigate common legal tactics used by large technology firms to delay implementing legal provisions that inconvenience them. Third, the realization of the right to constructive optimization depends on strong enforcement mechanisms, which, given the persistent lack of institutional resources and the overall complexity of the digital environment, has proven a long-standing challenge for competent authorities. Finally, if we want to increase the bargaining and contestation power of individuals and collective interest groups, they should have access to the prerequisite financial and political resources.

That being said, our current proposal is clear in its aspirations, and this clarity, we believe, should suffice as guidance for identifying those measures and environmental conditions that are conducive to a healthy, digital public sphere and those that are not. As such, it can already inspire future policy discussions, including those surrounding a potential Digital Fairness Act, and the interpretation and operationalization of the DSA (see below). Regulatory authorities tasked with the guidance and enforcement of EU technology legislation, such as the Digital Service Coordinators, the European Board for Digital Services, or the newly established EU’s AI Office, could take inspiration from this proposed direction when operationalizing the provisions of the DSA. And given the political interference exerted by Big Tech players, an alternative vision of the future might be more necessary now than ever. Of course, this should not withhold existing players and market entrants from reflecting and incorporating components of our proposal to embody a more inclusive ideation of tech governance. Similarly, self-regulatory efforts, like codes of conduct and the practical measures contained within them, could use our rights as an evaluative benchmark (see also Article 45 DSA). Whether these ambitions will be realized, ultimately depends upon the political goodwill and traction of policymakers, regulators, and platform operators. In the next section, however, we demonstrate that insofar as this goodwill exists, a right to constructive optimization could add much-needed substantive guidance to the primarily procedural approach of the DSA.

### A Revised Look at the DSA Through the Lens of Constructive Optimization

To meet the right to constructive optimization’s most ambitious objectives, legislative change would certainly be required. While we realize that it is unlikely that the DSA will be amended anytime soon, in the following, we will illustrate how the right to constructive optimization can nonetheless function as an interpretive lens and result in a more public-facing operationalization of the DSA’s recommender provisions.

#### Recommender Transparency

The DSA’s recommender provisions give users a right to know why they receive certain information (Article 27 DSA). Less clear and subject to interpretation is what information precisely this entails. The DSA speaks of “criteria which are most significant in determining the information suggested” and the “reasons for the relative importance of those parameters.” “Parameters” can refer to technical parameters in the sense of numerical values and their weights. Parameters, however, can also refer to the market logic behind a recommender, the extent to which it prioritizes personal content over news, which aspects of the personal profile the recommendation is matched with, the efforts a platform has undertaken to ensure the quality and diversity of the pool of information the recommender draws on, and what has been done to make sure to diversity and include content that reflects specific interests of marginalized groups. In other words, the right to constructive optimization can help to concretize users’ information needs and inform a teleological interpretation of Article 27 DSA.

#### Risk Assessments: Performance and Mitigation

The DSA obliges VLOPs to take into account the “design of their recommender systems” (Article 34 DSA). Again, the design of recommender systems can be interpreted narrowly in the sense of the models and how they have been developed, tested, and trained. We argue in favour of a broader interpretation, including the operational and managerial decisions that have guided the design choices; how public interests or the preferences of users have weighed into the decision; the extent to which users, including marginalized or vulnerable groups, have been included actively in the development and testing of the model; cooperation with researchers; or investment in experimentation with alternative, more diverse recommendation algorithms were made. Similarly, when evaluating risk sources, the “data-related practices of the provider” (Article 34 (2)(e) DSA) should be viewed solely not only in light of the right to privacy and respect for personal data but also in terms of efforts to ensure the quality of training and testing data, to address biases, increase the diversity of the content pool of recommended information, or give users agency over their profiles. Moreover, following a socio-technical perspective, risk assessments can only be fruitful if each evaluative component is viewed as part of a dynamic and interactive ecosystem rather than a static component that operates within a vacuum. For example, evaluating a recommender system might only make sense when viewed in relationship to existing content moderation policies and other (value-laden) practices, such as choices made during the data collection and curation phase.

Also, insofar as providers of VLOPs fail to demonstrate their compliance with standards of inclusivity and constructive optimization, this can create a strong indicator of a systemic risk in the sense of Art. 34 (1)(b) DSA. For example, if VLOP providers cannot demonstrate how they tried to engage with and listen to the diverse needs of citizens, and marginalized communities in particular, nor put in place, mechanisms of accountability and contestability, their operations threaten a well-functioning digital public sphere. In a similar way, a right to constructive optimization can guide the choice for adequate risk mitigation measures. This is effectively what the European Commission had already done for the role of recommender systems in the context of elections. The need for VLOPs to incorporate meaningful user-agency during election time was justified by the fact that “[o]nline platforms and search engines have become important venues for public debate and for shaping public opinion and voter behaviour” (European Commission 2024a, para 3.2.1. (27)(d)). In response and in light of the risk recommenders pose to electoral processes, the Commission noted that these systems should be designed and adjusted to give users meaningful choices and control over their feeds, with due regard to media diversity and pluralism (European Commission, 2024a).

#### A Right to Complain About the Lack of Constructive Optimization

As a final point, it is also worthwhile referring to Article 53 of the DSA, which confers onto users “the right to lodge a complaint against providers of intermediary services alleging an infringement of [the DSA].” Acknowledging a right to constructive optimization can assist users in making their claims concrete and substantiate a reversal of the burden of proof. Put simply, a right to constructive optimization would legally anchor citizens’ ability to complain about the unresponsible exercise over the recommender’s public function.

## Conclusion

Rather than view recommenders as a proprietary technology, the governance of which, is left to the sole discretion of VLOP providers, we have argued that these socio-technical systems perform an essential public dimension. Recommendations affect people in their access to social, economic, and political opportunities, including fundamental rights. Despite their centrality within the digital public sphere, users and affected non-users have little democratic agency as to how their living conditions are shaped by VLOPs and the technologies they employ. Users and affected non-users are placed before a take-it-or-leave-it situation. Any benefit they might derive from recommender technologies typically occurs under design and control conditions that favour the commercial interests and objectives of VLOP providers.

In this paper, we propose a paradigm shift and suggest that people have a right to constructive optimization, a right that offers people agency over the design, ideation, and development of recommender systems and the public functions they perform. We made concrete substantive suggestions on how VLOPs should promote people’s access to, and enjoyment of, the values of self-development and self-determination under ideals of inclusivity, political equality, reasonableness, and publicity. These ideals underscore the need for governance initiatives to pay particular attention to the structural inequalities faced by historically disadvantaged, marginalized, or otherwise rendered vulnerable individuals and groups. At the same time, they emphasize the general importance of having strong standards of accountability, representation, and public contestability concerning the value-laden choices and decisions that run across the socio-technical recommender stack whether these are technical, organizational, or political in nature. Though our proposal is intended as a long-lasting evaluative regulatory benchmark, we have demonstrated how the DSA can be a viable point of departure and how the right to constructive optimization can be used to further operationalize the DSA’s provisions toward more inclusive platform governance.

## Data Availability

No datasets were generated or analysed during the current study.

## Footnotes

1 The ideas outlined within this paper build upon prior work from the authors, and specifically their contributions in (Naudts et al., 2024a, b; Helberger et al., 2024b).
